# Online peer support for people with dementia: a scoping review protocol

**DOI:** 10.11124/JBIES-24-00343

**Published:** 2025-07-07

**Authors:** Esther Vera Loseto-Gerritzen, Catherine Talbot, Orii McDermott, Martin Orrell, Neil S. Coulson

**Affiliations:** 1 Institute of Mental Health, Mental Health and Clinical Neurosciences, School of Medicine, University of Nottingham, Nottingham, United Kingdom; 2 Department of Psychology, Bournemouth University, Poole, United Kingdom; 3 Nottingham Centre for Public Health and Epidemiology, Lifespan and Population Health, School of Medicine, University of Nottingham, Nottingham, United Kingdom; 4 The Nottingham Centre for Evidence-Based Healthcare: A JBI Centre of Excellence, University of Nottingham, Nottingham, United Kingdom

**Keywords:** cognitive impairment, dementia, digital, peer support, scoping review

## Abstract

**Objective:**

The objective of this scoping review is to gain insights into the different online peer support opportunities available for people with dementia.

**Introduction:**

People with dementia use a variety of online platforms for peer support, such as social networking sites or videoconferencing platforms. Online peer support can offer a variety of benefits, such as overcoming geographical barriers and the opportunity to choose a platform and mode of communication that suits a person’s needs and preferences. However, there is currently no synthesis of the different online peer support opportunities available to people with dementia.

**Eligibility criteria:**

Participants in this review will include people living with a self-reported diagnosis of any form of dementia. The concept is peer support through online platforms, while the context is online peer support that is accessible in English.

**Methods:**

The review will be conducted according to the JBI methodology for scoping reviews. A 3-step strategy will be used to search 4 data sources: scholarly and gray literature databases, Google search engine, social media and websites of dementia organizations, and consultations with people with dementia and professionals. The study selection and data extraction will be conducted by 2 reviewers independently, and a third reviewer will be consulted if needed. For the data extraction, a draft data extraction instrument will be used, following the recommendations of JBI. Data will be extracted on platform, online peer support, and study characteristics. The findings will be presented in tables and a narrative summary.

**Review registration:**

OSF https://osf.io/8jtqc

## Introduction

Dementia can have a significant impact on the person with the diagnosis and their family. A systematic review on dementia by Górska and colleagues^1^ states that common experiences include finding it difficult to adjust to the diagnosis and fear of an uncertain future. Furthermore, due to the nature of symptoms, people with dementia may lose confidence in their skills and abilities, including social interactions, and may fear embarrassment or judgment from others. People with dementia often face changes in their roles and responsibilities, changes in family dynamics, and a growing dependence on others. As a result, people with dementia can experience a loss of autonomy, independence, and their identity.^1^ People with young-onset dementia (YOD) (onset before the age of 65 years) experience additional, unique challenges, as they are more likely to be in paid employment at the time of symptom onset or diagnosis, and more likely to balance different roles and responsibilities toward dependent children or older parents.^2^

Psychosocial support is essential for helping people with dementia and their families adjust to the diagnosis, reduce disability, and improve independence and well-being. It focuses on providing “interaction between people to improve psychological and/or social functioning, including well-being and cognition, interpersonal relationships and everyday functional abilities.”^3^^(p.286)^ This links with the Social Health framework, which views health as a balance between the challenges that someone experiences because of their health, and the skills and abilities that they still have.^4^ In their European consensus work, Dröes *et al.*^5^ highlight the importance of incorporating social health into psychosocial interventions and health and social care services for people with dementia and their families. This is intended to support people in the 3 dimensions of social health: one’s capacity to “(1) to fulfill one’s potential and obligations, (2) manage life with some level of independence, and (3) participate in social activities.”^5^^(p.11)^

One way to support the social health of people with dementia is through peer support. Peer support—mutual support between people who have a similar health condition or life experience^6^—offers opportunities for new social interactions and activities. It also allows people to exchange experiences and advice around coping mechanisms and support services. Moreover, the reciprocal nature of peer support can increase autonomy, independence, and empowerment of people with dementia.^7^ Thus, peer support can be crucial in helping people to live as well as possible with dementia. This is important, as Hevink *et al.*^8^ indicate that people with dementia and their informal carers are often not satisfied with the information that professionals provide on living well with dementia.

Peer support can take place in both in-person as well as online settings. Research shows that people with dementia use a variety of online platforms for peer support, such as through blogs,^9^,^10^ discussion forums,^11^,^12^ social networking sites,^13^,^14^ and videoconferencing platforms.^15^ While people of any age may make use of online peer support, those of a younger age are more likely to engage with online platforms, including people with dementia.^16^ Peer support through these online platforms can offer a variety of benefits. Online peer support can overcome geographical barriers, which can be particularly important for those with rare forms of dementia, as it can be difficult for these groups to meet peers in their local area.^17^ Online platforms also make peer support accessible to those who are unable to travel to in-person settings, for example, due to the nature of their symptoms or a lack of accessible transportation.^18^

Furthermore, online platforms can offer different sizes and modes of communication and tend to be accessible on a variety of devices, including desktop computers and laptops, smart phones, or tablet computers. Text-based asynchronous communities (eg, via discussion forums or social media) tend to be relatively large, whereas videoconferencing platforms offer synchronous, audio-visual communication, often in smaller groups. The variety of online platforms available to people with dementia allows users to choose something that suits their needs and preferences. While there are many benefits, it is important to also acknowledge that there are challenges associated with online peer support, such as digital exclusion, exposure to misleading or harmful information, or harmful interactions.^19^

While there are several literature reviews on online peer support for informal carers of people with dementia,^20^-^22^ there is a lack of reviews on online peer support for people diagnosed with dementia. Consequently, there is currently no overview of what different online peer support opportunities are available to people with dementia. Accessible information about post-diagnostic support is important for people to adapt to their diagnosis and to maintain well-being.^8^ A scoping review found that the information needs of people with dementia and their informal carers are often not met. While this may be due to a lack of information, it is also because information is difficult to find and presented in a way that is not accessible.^23^ These findings are illustrated by our previous research into online peer support for people with YOD, which showed that many people were unaware that online peer support exists or where to find more information.^24^ Furthermore, our previous qualitative work showed that people with YOD sometimes felt hesitant to engage with online peer support groups because they were unsure of what to expect.^25^

These findings suggest that there is a need for a clear and accessible overview of online peer support opportunities for people with dementia, including its characteristics. Scoping reviews aim to provide a broad overview of what is known about a certain topic and use a broad scope of data sources, including both the scholarly and gray literature. Therefore, a scoping review can be a useful method to obtain insights into different online peer support opportunities for people with dementia.

## Review questions

- What online peer support is available for people with dementia who are English speakers?
- What online peer support is available specifically for people with YOD who are English speakers?
- What are the characteristics of online peer support for people with dementia who are English speakers?

## Eligibility criteria

### Participants

For this scoping review, we will consider adults with any type of self-reported dementia diagnosis. We will exclude studies that explore health care professionals’ engagement with online support groups for dementia and studies about online peer support groups solely aimed at carers (formal or informal). However, we will include studies on both carers and people with dementia.

### Concept

The concept for this scoping review is the availability and characteristics of online peer support groups and communities for people with dementia. Fortuna and colleagues^6^ define peer support as:

social and/or emotional support that combines expertise from lived experience that is delivered with mutual agreement by persons who self-identify as having or had mental health as well as other social, psychological and medical challenges to service users sharing similar challenges to bring about self-determined personal change to the service user.^(p.573)^

For this review, we define online peer support as communication via the internet between peers in an online environment that is designed to facilitate social contact.

Regarding the availability of online peer support, we aim to capture a broad range of online peer support opportunities. Therefore, we will also include online group interventions for people with dementia that do not have peer support as the primary outcome but that include peer support elements. Interventions that only include in-person or telephone-based peer support will be excluded.

For the characteristics of online peer support, we are interested in the type of platform, modes of communication (eg, synchronous vs asynchronous, or text-based vs audio-visual communication), levels of moderation, and group composition (eg, only people with dementia or both people with dementia and carers).

### Context

In this review, we will include online peer support opportunities for people with dementia in an international context, including groups and communities from any geographical setting that use the English language. This context will enable us to capture online peer support groups across diverse social and cultural settings. By focusing on groups and communities that speak English, we aim to generate a comprehensive overview of online peer support opportunities for people with dementia that can be helpful to a diverse and international audience.

### Types of sources

This scoping review will consider empirical studies of any design (eg, qualitative, quantitative, mixed methods) as well as gray literature sources that report on online peer support for people with dementia.

## Methods

We will conduct this review in accordance with the JBI methodology for scoping reviews^26^ and will use the Preferred Reporting Items for Systematic Reviews and Meta-Analyses extension for Scoping Reviews (PRISMA-ScR) checklist to report the review.^27^ This protocol has been registered in OSF: https://osf.io/8jtqc.

### Search strategy

A 3-step search strategy will be followed. As a first step, we conducted an initial search of at least 2 databases for title and abstract screening. An example of the MEDLINE (Ovid) search strategy is presented in Appendix I. Second, we will conduct a full search of the 4 data sources to be used in the review. These will include i) academic and gray literature databases; ii) the Google search engine; iii) websites, including social media websites and websites of UK-based or international dementia organizations; and iv) consultations with people with dementia as well as health and social care professionals working with people with dementia. We identified these sources in the work of Godin *et al.*^28^ who applied systematic search methods to the gray literature, including the Google search engine, websites, and expert consultations. By including academic and gray literature databases as well as website searches, we will ensure that no relevant publications are omitted. This will also ensure we do not miss any online peer support opportunities for people with dementia that are not included in the findings from academic or gray literature databases. In the final step of the search strategy, we will hand-search the reference lists of sources that have been selected for full-text review. We will limit the search strategy to sources in English. All searches will be conducted by a trained librarian (NT).

An overview of how the different data sources will be searched is presented as follows.

#### Data source 1: Scholarly and gray literature databases

The scholarly databases will include MEDLINE (Ovid), CINAHL Plus (EBSCOhost), Cochrane Library, PsycINFO (Ovid), Scopus, and Web of Science Core Collection. The gray literature databases will include ProQuest Dissertations and Theses and MedNar. We will not apply limitations on the year of publication. We will also search Google Scholar. For Google Scholar we will only screen the first 10 pages, in line with Godin *et al.’s*^28^ systematic search using the Google search engine.

#### Data source 2: Google search engine

We will search the Google search engine using different combinations of the search terms listed in Appendix I. Due to the large amount of search results that are likely to be generated from the Google search, we will only review the first 10 pages, following the example of Godin *et al*.^28^ A librarian (NT) will conduct the Google and website (data source 3) searches with the internet browser in incognito mode, which does not track cookies and is completely independent of any previous internet history to avoid influencing the search results.

#### Data source 3: Websites

We will search social media websites and the websites of UK-based and international dementia organizations, following the example of Monnet and colleagues.^29^ First, the research team will compose a list of relevant organizations and websites, in collaboration with patient and public involvement (PPI) members and professionals working with people with dementia. Second, the librarian (NT) will search the websites using different combinations of the search terms (Appendix I). If possible, the websites will be searched using the search bar, before hand-searching them to ensure that no relevant information is missed. Websites without a search bar will be hand-searched and the “search within site” function of Google will also be used for these websites if necessary.

#### Data source 4: Consultations with people with dementia and professionals

Through PPI and stakeholder consultations, we will ask people with dementia and their families, as well as health and social care professionals, to review the findings of the database, Google search engine, and website searches, and add any other online peer support groups that were omitted. We will identify people with dementia and their families through existing PPI networks at the University of Nottingham and Bournemouth University. We will identify professionals through the authors’ networks and dementia organizations.

### Study selection

We will import the results from the academic and gray literature database searches into Covidence (Veritas Health Innovation, Melbourne, Australia), after which we will remove the duplicates. Covidence is a web-based software that can be used to manage the study selection phase among multiple reviewers. Two reviewers will independently select the studies. The reviewers will first screen a sample of 10% of the total items to be reviewed, to improve agreement between the reviewers. Any disagreement between the reviewers and any studies that the reviewers are unsure of will be resolved through discussion or the involvement of a third reviewer if needed. First, the reviewers will review the titles and abstracts. Second, they will screen the full text of potentially relevant titles and abstracts against the eligibility criteria. They will then enter the results from the Google search, website search, and consultations with people with dementia and their families and health and social care professionals into a Microsoft Excel (Redmond, Washington, USA) spreadsheet. These results will be reviewed against the eligibility criteria.

### Data extraction

The same 2 reviewers will independently extract the data, and any disagreements will be resolved the same way as for study selection. First, the reviewers will extract data on a 10% sample and discuss whether the data extraction form or process needs any adjustments. The review team will extract data using a draft data extraction instrument, which they adapted from the JBI standardized tool (see Appendix II). We will contact authors or contact persons of online peer support groups for additional information if needed, with up to 2 reminders in case of no response.

### Data analysis and presentation

We will present the results of this review in a PRISMA flow diagram.^30^ We will synthesize the findings of the database, Google search engine, website searches, and PPI consultations in tables and a narrative summary. The findings of this review will provide a broad overview of the online peer support opportunities that are available for people with dementia who understand English. Dementia organizations and health and social care professionals can use these findings to signpost people with dementia to online peer support. The findings of this review will help determine whether any gaps exist in the current availability and accessibility of online peer support for people with dementia, which can inform future research into this area.

## Acknowledgments

Naomi Thorpe, senior information specialist at the Institute of Mental Health for the Library and Knowledge Services of the Nottinghamshire Healthcare NHS Foundation Trust, for developing the search strategy and conducting the searches.

## Funding

This review is funded by the NIHR Applied Research Collaboration East Midlands (ARC EM). The views expressed are those of the authors and not necessarily those of the NIHR or the Department of Health and Social Care.

## Appendix I: Search strategy

### MEDLINE (Ovid)

Search conducted: July 12, 2024

**Table:**

#	Search term(s)	Records retrieved	Concepts
1	exp Dementia/	215,255	Dementia
2	(dement* or alzheimer*).tw,kf.	298,248	
3	or/1-2	340,102	
4	exp Internet/ or exp Online Social Networking/ or exp Social Media/ or exp Videoconferencing/	103,356	Online peer support
5	(digital or internet or “social media” or “app based” or “web based” or app-based or web-based or videoconferenc* or “video conferenc*”).tw,kf.	346,323	
6	((online or virtual) adj2 ("support group*” or “support commun*” or group* or commun* or self-help or “self help” or selfhelp or forum*)).tw,kf.	9977	
7	("discussion forum*” or “bulletin board*” or “chat room*” or “computer-mediated support*” or “message board*”).tw,kf.	2044	
8	((internet or “web-based” or “web based”) adj2 ("support group*” or “support commun*”)).tw,kf.	161	
9	((peer or peers or peer-to-peer or mentor* or counsel*) adj2 (support* or led or lead* or deliver* or run* or held or direct* or online or “on line” or forum*)).tw,kf.	20,405	
10	or/4-9	418,278	
11	3 and 10	3278	Dementia AND online peer support
12	limit 11 to English language	3170	Dementia AND online peer support AND English language

## Appendix II: Draft data extraction instrument

**Table:**

Authors/group name	Year of publication or when group was founded	Platform	Aims/purpose	Population and sample size/target audience and group size (eg, people with dementia, people with young-onset dementia, or people with dementia and informal carers)	Methodology	Intervention type or mode of communication (eg, synchronous or asynchronous communication, verbal or text-based communication)	Key findings
							
							
